# Estrogen is involved in hemangioma regression associated with mast cells

**DOI:** 10.1186/s13023-018-0928-x

**Published:** 2018-10-19

**Authors:** Fang Hou, Yuemeng Dai, Chun-Yang Fan, James Y. Suen, Gresham T. Richter

**Affiliations:** 1 Department of Pediatric Surgery, Sichuan Academy of Medical Sciences & Sichuan Provincial People’s Hospital, Chengdu, 610072 China; 2 School of medicine, University of Electronic Science and Technology of China, Chengdu, 610072 China; 3 Center for the Investigation of Congenital Aberrancies of Vascular Development, Little Rock, AR USA; 4 Department of Pathology, University of Arkansas for Medical Sciences, Little Rock, AR USA; 5 Department of Otolaryngology, University of Arkansas for Medical Sciences, Little Rock, AR USA; 6 Division of Pediatric Otolaryngology, Arkansas Children’s Hospital, 1 Children’s Way, Little Rock, AR 72202 USA

**Keywords:** Infantile hemangioma, Mast cells, Estradiol, Estrogen receptor

## Abstract

**Background:**

Estrogen plays a role in infantile hemangioma (IH) development, but the underlying mechanism remains unclear. This study aimed to assess estrogen and estrogen receptor (ER) localization and expression levels in IH. In addition, the unexpected relationship between mast cells (MCs) and estrogen in human IH was discussed.

**Methods:**

IH (*n* = 29), vascular malformation (VMs, *n* = 33) and normal skin (*n* = 15) specimens were assessed. IH was classified into proliferative (*n* = 9; age, 3.56 ± 1.01 months), early involuting (*n* = 10; age, 8.90 ± 2.69 months) and late involuting (*n* = 10; age, 20.10 ± 4.93 months) groups. Estradiol (E2), ER-a, ER-β, and tryptase (MC marker) levels were determined immunohistochemically and/or by double immunofluorescence staining. Quantification and localization of tryptase, ER-a, and E2 were assessed for each specimen.

**Results:**

ER-a, E2, and tryptase were expressed in the cytoplasm and nucleus of MCs in IH. The IH specimens showed significantly more tryptase, ER-a, and E2 positive MCs (30.6 ± 12.7, 9.7 ± 5.6, and 19.8 ± 8.7 cells/high-power field [HPF], respectively) compared with VM specimens (9.0 ± 9.8, 1.5 ± 2.4, and 2.5 ± 4.1 cells/HPF, respectively) and normal skin (6.1 ± 8.5, 0.5 ± 1.2, and 1.9 ± 3.4 cells/HPF, respectively). Proliferating IH displayed fewer E2 positive MCs (14.0 6.3 cells/HPF) compared with early (22.3 ± 10.2 cells/HPF, *P* = 0.023) and late (22.4 ± 6.8 cells/HPF, *P* = 0.006) involuting specimens. In addition, proliferating IH showed fewer tryptase positive MCs (24.7 ± 10.8 cells/HPF) compared with early involuting specimens (35.7 ± 15.3 cells/HPF, *P* = 0.043). All IH specimens were ER-a positive and ER-β negative.

**Conclusions:**

E2 and ER-a are expressed on MCs and not on IH endothelial cells. Furthermore, activated MCs may be involved in IH regression.

## Background

Infantile hemangioma (IH) is the most common benign tumor of infancy, with an incidence estimated at 4.5%, which is higher in the female, caucasian race and low birth weight infants [1, 2]. The natural history of IH is peculiar. It appears at birth or within the first weeks of life and it undergoes a rapid proliferation phase that lasts until the 12th months of life. Then, IH undergoes an involuting phase with a complete regression in 90% of cases by age 4 years [1]. IH’s life cycle can be envisioned as a play in three overlapping acts, including proliferation (proliferating phase), early involution (early involuting phase) and late involution (late involuting phase) [3]. Histologically, the proliferating phase is characterized by an abundance of rapidly dividing endothelial cells and mast cells (MCs). During early involuting phase, apoptotic bodies and MCs increase in number and capillaries begin to diminish. As an IH progresses to the late involuting phase, it is characterized by slowly diminishing vascular channels, MCs, and endothelial cells, which are gradually replaced by fibrofatty tissues [3–5]. However, there is no sharp dividing line between proliferation and involution. When involution patterns are more frequent than proliferation ones, IH exhibits spontaneous regression. Although the pathophysiology of proliferation and involution processes in IH remains unclear, imbalanced levels of angiogenic and antiangiogenic factors may contribute to these phenomena [3, 4].

There is also mounting evidence linking estrogens with IH. For instance, IH incidence is up to three times more common in girls than in boys [1–6]. Previous findings revealed that serum estradiol (E2) levels in IH patients are fourfold higher than those of healthy children [7]. Moreover, estrogen promotes the proliferation of IH’s vascular endothelial cells synergistically with vascular endothelial growth factor (VEGF) in vitro [8]. However, little is known about the presence and mechanisms of estrogen and estrogen receptor (ER) in the pathological process of IH.

Several studies confirmed that E2 modulates MC migration and degranulation [9–11]. Moreover, MCs displayed ER on their surface, which can be activated by the binding of E2 [9]. Although MCs (tryptase positive) are found throughout the whole life cycle of IH [12], ER or E2 expression on MCs in IH remains unexplored.

We assume that MC precursors migrate into the IH tissue attracted by E2 and then, MCs undergo maturation and activation by E2 binding to ER expressed on MCs. Therefore, this study aimed to assess E2, ER, and tryptase expression profiles in MCs in the proliferative, early involuting and late involuting phases of IH. Furthermore, the role of MC activation by E2 binding to ER in IH was discussed.

## Methods

### Specimens

This study was approved by the Institutional Review Board of the University of Arkansas for Medical Sciences. After obtaining informed consent, fresh surgical IH tissue specimens were obtained from 29 patients; vascular malformations (VMs) were obtained from 33 patients (12 lymphatic malformations, 8 venous malformations, 13 arteriovenous malformations); and normal skin with subcutaneous tissue samples were obtained from 15 patients. Specimens were from untreated patients and then, they have been fixed with 10% buffered formalin, paraffin embedded, serially cut into 4 μm sections, and stained with hematoxylin-eosin (H&E).

Histologic examination by a pathologist with experience in vascular anomalies (VAs) confirmed the diagnosis for each patient at the time of resection. All IH specimens were confirmed by histological assessment and positive staining for glucose transporter protein-1 (GLUT-1), a marker that immunohistochemically distinguishes IH from other VAs [3].

### Immunohistochemistry

After deparaffinization and rehydration, the sections were heated to 97 °C for 20 min in the presence of an antigen retrieval solution (CITRA, pH 6.0; Invitrogen, Carlsbad, CA) and cooled for 30 min. To block endogenous peroxidase activity, the sections were incubated with hydrogen peroxide for 10 min and washed with phosphate-buffered saline (PBS, pH 7.4; Sigma-Aldrich, St Louis, MO). After pre-incubation with 2% nonfat milk for 30 min at room temperature, the sections were incubated with primary antibodies targeting Glut-1 (mouse monoclonal antibody; Abcam, Cambridge, MA; 1:200), estradiol (E2, rabbit polyclonal antibody; Millipore, Billerica, MA; 1:2000), tryptase (mouse monoclonal antibody; Abcam, Cambridge, MA; 1:200), ER-a (mouse monoclonal antibody; Thermo Fisher Scientific, Waltham, MA; 1:100), and ER-β (rabbit polyclonal antibody; Invitrogen, Carlsbad, CA; 1:50), respectively, for 20 h at 4 °C. Negative controls were incubated without primary antibodies. After washing with PBS, the sections were sequentially incubated with primary antibody enhancer (Thermo Fisher Scientific, Waltham, MA; 10 min) and horseradish peroxidase polymer (Thermo Fisher Scientific, Waltham, MA; 15 min) at room temperature. Next, the sections were incubated with diaminobenzidine (DAB; Thermo Fisher Scientific, Waltham, MA) for 3 min at room temperature, and counterstained with hematoxylin for 30 s. After dehydration by graded alcohol solutions and cleaning with xylene substitute, the sections were mounted (with Permount; Thermo Fisher Scientific, Waltham, MA) and coverslipped.

### Double immunofluorescence labelling

After deparaffinization and rehydration, the sections were heated to 97 °C for 20 min in the presence of an antigen retrieval solution (CITRA, pH 6.0; Invitrogen, Carlsbad, CA) and cooled for 30 min. To block the endogenous peroxidase activity, all sections were incubated with hydrogen peroxide for 10 min and washed with PBS-Tween. The sections were then pre-incubated with 2% nonfat milk for 30 min at room temperature, followed by incubation with a mixture of two primary antibodies targeting estradiol and tryptase, respectively, overnight at 4 °C. After washing with PBS-Tween, the sections were incubated with a mixture of two fluorescent conjugated secondary antibodies (FITC conjugated Goat anti-Rabbit and Texas Red conjugated Goat anti-Mouse, Invitrogen, Carlsbad*,* CA) diluted in PBS, for 60 min at room temperature. Counterstaining was performed with 4′,6-diamidino-2-phenylindole (DAPI, Invitrogen, Carlsbad, CA) for 20 min at room temperature. The sections were then washed in PBS-Tween and coverslipped using an anti-fade fluorescent mounting medium*.* After sealing with nail polish, the specimens were stored in the dark at 4 °C.

### Mast cell counts (MCC) and statistical analyses

MCs expressing tryptase, E2 and ER-a in all specimens were counted in 9 random high power fields (HPF, 400X) per sample. Counts were expressed as mean ± SD. Student’s t-test was used to compare group pairs. *P* < 0.05 was considered statistically significant.

## Results

**Fig. 1:**
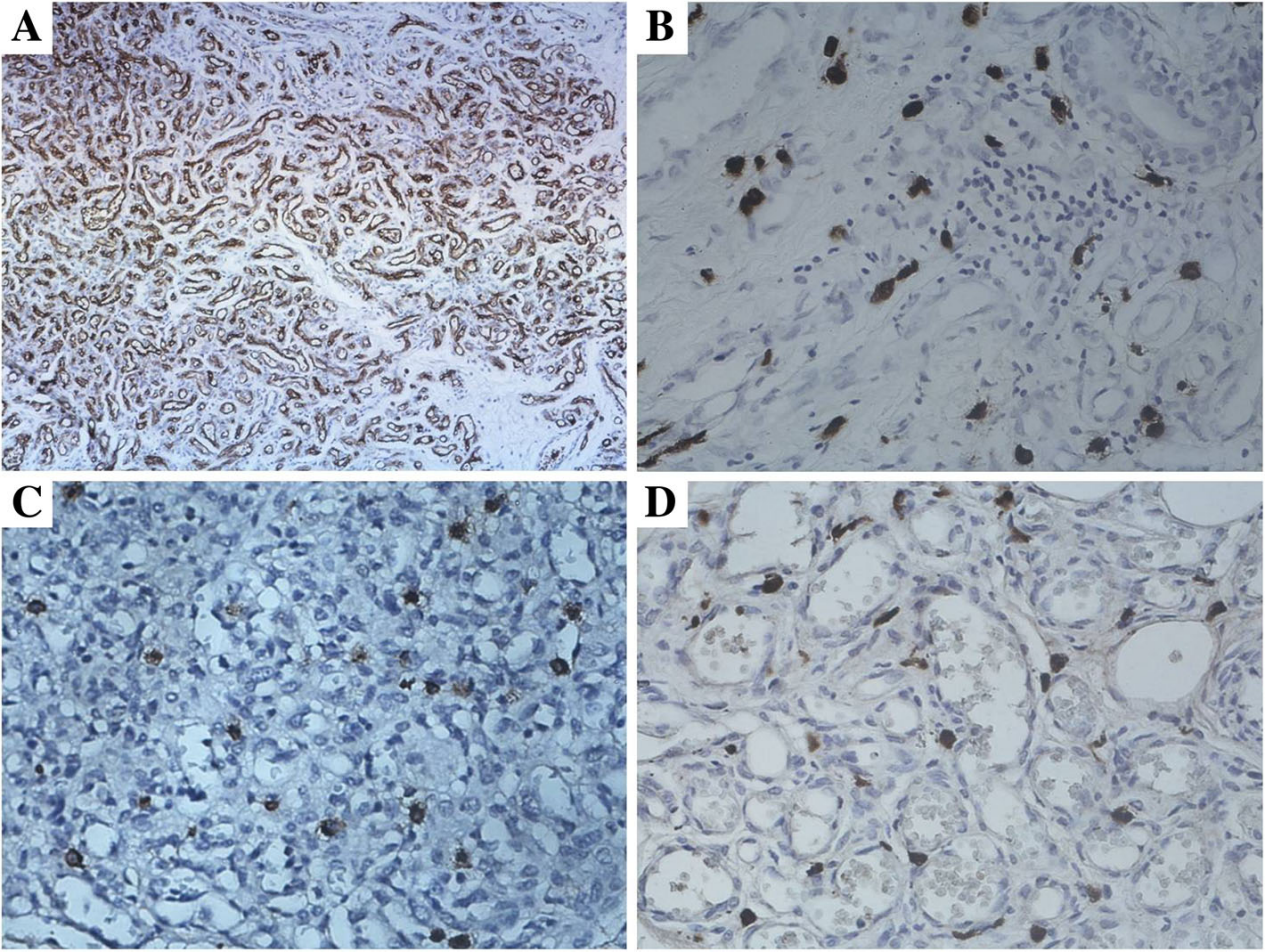
Immunostaining for Glut-1, E2, tryptase and ER-a in IH. **a** All IH specimens were positive for GLUT-1, which was localized in the cytoplasm of IH endothelial cells (IHC, × 100). Large numbers of tryptase (**b**), ER-a (**c**), and E2 (**d**) positive cells were observed in all IH specimens, specifically in the cytoplasm and nucleus of MCs, but not in IH endothelial cells (IHC, × 400)

All IH specimens were confirmed by histology and GLUT-1 positive staining (Fig. 1-a). Based on histological characteristics and patient ages, the IH specimens (*n* = 29) were divided into 3 groups: proliferative (*n* = 9; 3.56 ± 1.01 months), early involuting (*n* = 10; 8.90 ± 2.69 months) and late involuting (*n* = 10; 20.10 ± 4.93 months) (Table 1).

**Table 1:** Division of IH specimens (*n* = 29) into 3 groups

Proliferative phase, *n* = 9		Early involuting phase, *n* = 10		Late involuting phase, *n* = 10	
Age: M	Sex	Age: M	Sex	Age: M	Sex
2	F	6	F	15	F
2	F	6	M	15	F
3	M	7	M	15	F
4	F	7	M	18	F
4	F	7	M	18	M
4	F	9	M	20	M
4	M	10	F	22	F
4	F	12	F	24	M
5	F	12	M	24	F
		13	F	30	F
3.56 ± 1.01^a^		8.90 ± 2.69^a^		20.10 ± 4.93^a^	

The proliferative, early involuting, and late involuting phase groups were constituted

^a^Values are mean ± SD months

### Immunohistochemical findings

**Fig. 2:**
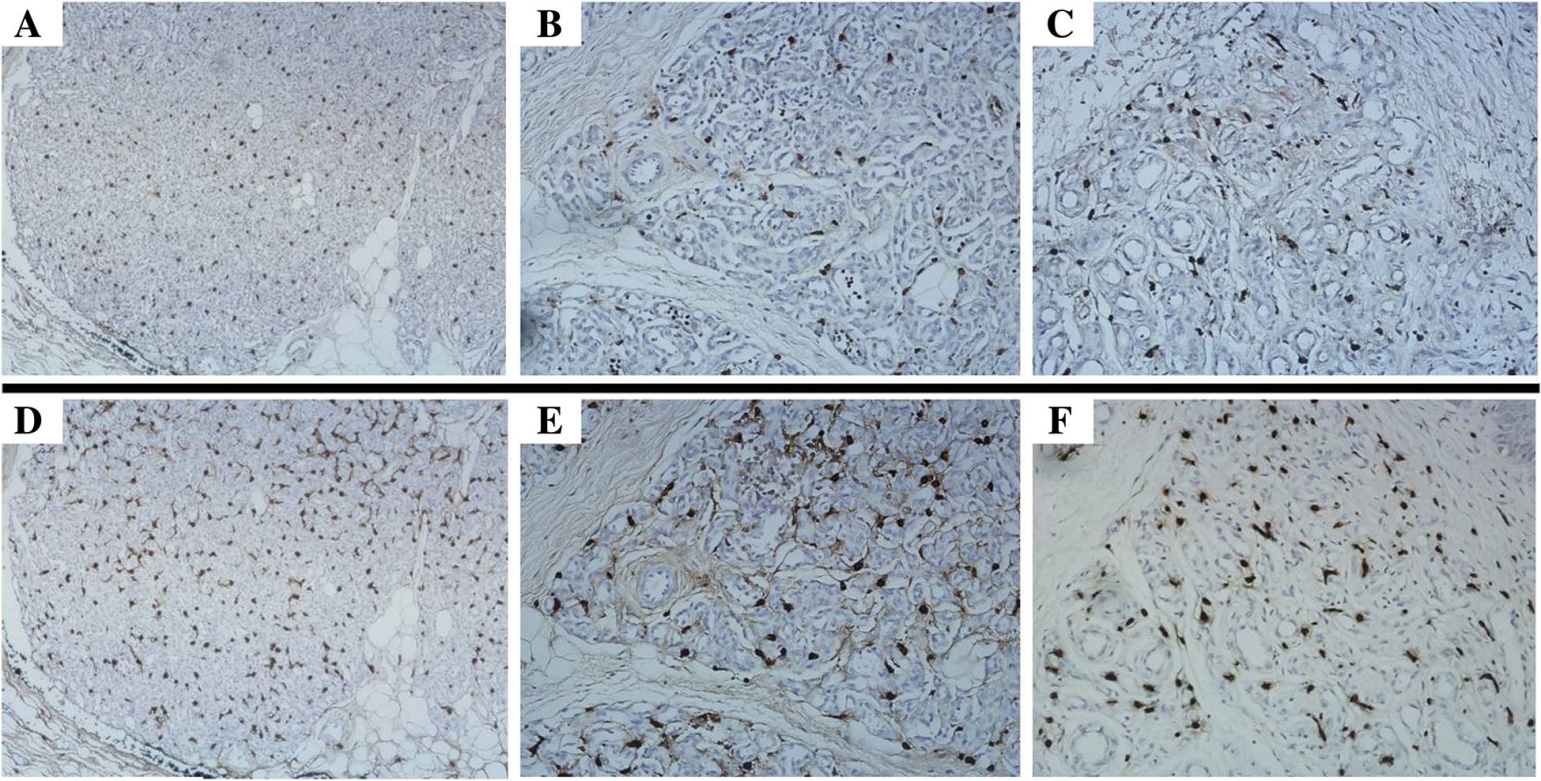
Immunostaining for E2 and tryptase in IH. Large numbers of E2 (**a**, IHC, × 100; **b** and **c**, IHC, × 200) and tryptase (**d**, IHC, × 100; E and F, IHC, × 200) positive MCs were found within all IH specimens. IH in the proliferative phase (**a, b, d**, and **e**) contained fewer E2 and tryptase positive MCs compared with early involuting phase specimens (**c** and **f**) (IHC, × 200)

To detect MCs in the specimens, immunohistochemical analysis for tryptase, a specific and sensitive MC maturation marker [10, 12], was performed. Large numbers of tryptase positive cells were observed within all IH specimens. Tryptase was specifically localized in the cytoplasm of MCs (Figs. 1 and 2). Average total MCC in proliferative, early involuting, and late involuting phases were 24.7 ± 10.8, 35.7 ± 15.3, and 30.7 ± 9.9 cells/HPF, respectively. These findings suggested that IH in the proliferative phase contained significantly fewer tryptase positive MCs compared with early involuting specimens (*P* = 0.043) (Table 2). There was no significant difference between proliferative and late involuting phase.

**Table 2:** Positive MCC in IH: IH in the proliferative phase showed fewer tryptase positive MCs compared with early involuting phase specimens

Proliferative phase, Cells/HPF	Early involuting phase, Cells/HPF	Late involuting phase, Cells/HPF
21	34	23
16	29	47
15	20	26
20	23	23
25	45	33
35	24	29
21	64	29
49	20	49
20	53	20
	45	28
24.7 ± 10.8^a^	35.7 ± 15.3^a^	30.7 ± 9.9^a^

Proliferative versus early involuting, *P* = 0.043

^a^Values are mean ± SD Cells/HPF

To assess whether ER is expressed in IH, we performed immunohistochemistry using anti-ER-a and ER-β antibodies. All IH specimens were ER-a positive and ER-β negative. In particular, ER-a was localized in the cytoplasm and nucleus of MCs but not in endothelial cells (Fig. 1-c). Average total ER-a positive cell counts per HPF in the proliferative, early involuting, and late involuting phases were 7.9 ± 4.3, 9.7 ± 5.0, and 11.2 ± 6.9, respectively. ER-a positive cell counts were not significantly different among the IH groups (*P* > 0.05) (Table 3).

**Table 3:** Positive MCC in IH: ER-a positive cell counts were not different among IH groups

Proliferative phase, Cells/HPF	Early involuting phase, Cells/HPF	Late involuting phase, Cells/HPF
14	11	10
9	9	18
2	11	11
6	8	9
3	6	18
4	9	7
9	18	6
12	1	0
12	17	9
	7	24
7.9 ± 4.3^a^	9.7 ± 5.0^a^	11.2 ± 6.9^a^

No difference between these IH groups, *P* > 0.05

^a^Values are mean ± SD Cells/HPF

**Fig. 3:**
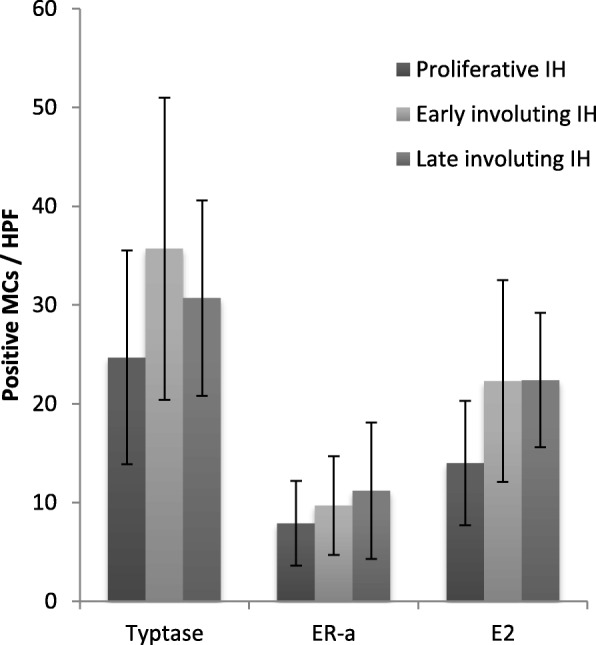
Dual staining for E2 (green, A) and tryptase (red, B) of IH sections. **a**, **b** Multiple cells (white arrowheads) showed strong immunoreactivity for E2 (**a**) and tryptase (**b**) (IHC, × 200). **c** Cell nuclei were counterstained with DAPI (blue, C) (IHC, × 200). **d** Merged image showing multiple MCs (white arrowheads) dually stained for E2 and tryptase (IHC, × 200)

To detect cells expressing estrogen, immunohistochemistry staining for E2 was performed. All IH specimens were positive for E2, which was localized in the cytoplasm and nucleus of MCs, but not in endothelial cells (Figs. 1 and 2). Average total E2 positive cell counts in the proliferative, early involuting, and late involuting phases were 14.0 ± 6.3, 22.3 ± 10.2, and 22.4 ± 6.8 cells/HPF, respectively, showing fewer E2 positive MCs in the proliferative phase compared with early and late involuting phase specimens (*P* = 0.023 and *P* = 0.006, respectively) (Table 4, Fig. 3).

**Table 4:** Positive MCC in IH: IH in the proliferative phase contained fewer E2 positive MCs compared with early and late involuting phase specimens

Proliferative phase, Cells/HPF	Early involuting phase, Cells/HPF	Late involuting phase, Cells/HPF
13	38	17
10	20	35
5	16	16
7	20	28
18	19	28
18	16	20
12	25	17
25	9	15
18	42	20
	18	28
14.0 ± 6.3^a^	22.3 ± 10.2^a^	22.4 ± 6.8^a^

Proliferative versus early involuting, *P* = 0.023

Proliferative versus late involuting, *P* = 0.006

^a^Values are mean ± SD Cells/HPF

IH specimens contained significantly more tryptase, ER-a, and E2 positive MCs (30.6 ± 12.7, 9.7 ± 5.6, and 19.8 ± 8.7 cells/HPF, respectively) compared with VM specimens (9.0 ± 9.8, 1.5 ± 2.4, and 2.5 ± 4.1 cells/HPF, respectively) and normal skin (6.1 ± 8.5, 0.5 ± 1.2, and 1.9 ± 3.4 cells/HPF, respectively). (*P* < 0.05) (Table 5).

**Table 5:** Tryptase, ER-a, and E2 positive mast cells in IH, VM, and normal skin samples

Specimens	Typtase positive, mean ± SD cells/HPF	ERa positive, mean ± SD cells/HPF	E2 positive, mean ± SD cells/HPF
IH (n = 29)	30.6 ± 12.7	9.7 ± 5.6	19.8 ± 8.7
VMs (*n* = 33)	9.0 ± 9.8	1.5 ± 2.4	2.5 ± 4.1
Normal Skin (*n* = 15)	6.1 ± 8.5	0.5 ± 1.2	1.9 ± 3.4

IH contained significantly more Typtase, ERa and E2 positive mast cells (*p* < 0.05)

### Double immunofluorescence staining

**Fig. 4:**
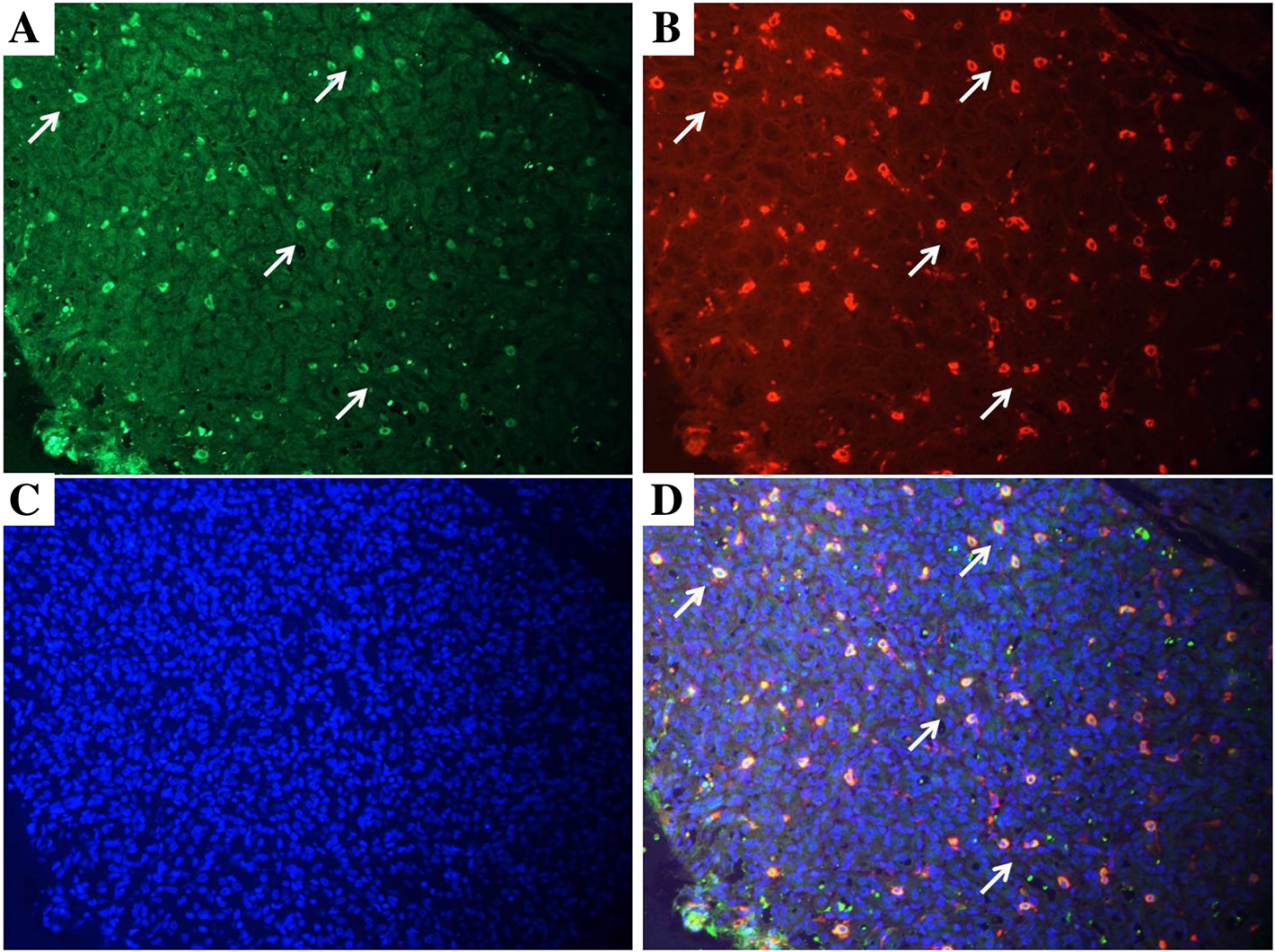
Positive MCC in IH: General patterns of positive MCC in different phases of IH (mean±SD).

To confirm that E2 positive cells were localized in MCs, double immunofluorescence staining for tryptase (red) and E2 (green) was performed under identical conditions for all IH sections. E2 tryptase positive cells in all IHs (Fig. 4).

## Discussion

In the past 30 years, several studies have suggested that estrogens and ER may play important roles in the development of IH [6–8]. However, little is known about the presence and the mechanisms of estrogens and ER in the pathological process of IH. There are three major forms of physiological estrogens: estrone (E1), estradiol (E2, or 17β-estradiol), and estriol (E3), among which, E2 is the dominant and the most effective [13]. Estrogens act through two types of receptors, including nuclear (ER-a and ER-β) and cell-membrane (GPR30 and ER-X) receptors. To date, however, few studies have assessed ER-a expression in IH tissues, indicating that estrogen may play a potential role in the development of IH [7, 14, 15]. In our study, MCs in IH showed strong positive staining for E2, ER-a, and tryptase, conversely E2 and ER-a were not expressed in endothelial cells.

In literature, it has been reported that the MC number fluctuates during the growth IH phases [16–19] and that highest MC count was observed during the involuting phase [18], consistent with our findings. In fact, IH in the proliferative phase contained fewer tryptase positive MCs compared with early involuting phase samples. However, Hasan et al. revealed that although the total number of MCs is highest during the early involuting phase of IH, the proportion of proliferating MC is highest during the proliferative phase and lowest during the late involuting phase [19]. The mechanisms underlying MC presence and MC count alterations in IH remain unclear.

Several studies have demonstrated that E2 attracts MCs by modulating the expression of chemokine receptors on their surface and activates MCs via binding to ER-a [9–11]. Our findings that E2 exists in IH and binds to ER on MCs may provide the first evidence for E2 involvement in the regulation of MC migration in IH. E2 also has an influence on MC maturation. A previous study reported that E2 significantly upregulated the expression of tryptase in the human immature mast cell line HMC-1 cells [10]. Tryptase, as a MC maturation marker, is the most abundant secretory granule-derived serine proteinase contained in MCs [12]. Two morphologic forms of MCs are known in IH, including immature (mainly in the proliferative phase) and mature (predominantly during involution) [3]. In this study, the tryptase positive MCs were highest in the early involuting phase and lowest in the proliferative phase. Our results suggest that during the proliferative phase, MC precursors migrate into the IH tissue, attracted by E2, then undergo maturation and act during IH involution.

MCs may be activated through various mechanisms. Binding of E2 (at physiological concentrations) to a membrane ER-a initiates a rapid onset and progressive influx of extracellular Ca2+, which results in MC activation [9]. This type of E2 modulation in MCs requires ER-a, but not ER-β [9, 10]; consistently, only ER-a was expressed on MCs in IH, as shown above. In our study, MCs in IH showed strong positive staining for E2, indicating that E2 positive MCs are activated. To our knowledge, this finding provides the first evidence for the presence of activated MCs in IH.

MCs are known to secrete proangiogenic and anti-angiogenic modulators once activated, e.g. proangiogenic factors, such as histamine, tryptase and chymase, which induce angiogenesis [20–22]. Furthermore, MCs in the proliferating phase of IH express the angiogenic factor fibroblast growth factor (FGF)-2, which promotes endothelial cell proliferation in IH [18, 23]. Anti-angiogenic cytokines, such as interferon (IFN)-a, IFN-β, IFN-γ and transforming growth factor (TGF)-β [18]. IFN-a and IFN-β can down-regulated the angiogenic factor FGF-2 [23, 24], while IFN-γ inhibits the mitogenic effect of VEGF [25] (FGF-2 and VEGF are significant angiogenic factors in proliferating IH) [26]. TGF-β plays a crucial role in vascular remodeling and maturation during the angiogenic process [27]. It has been shown that TGF-β inhibits endothelial cell proliferation and stimulates extracellular matrix deposition, while lack of TGF-β may increase proliferation in endothelial cells [12, 27]. Ultrastructural evidence suggests that adjacent cells (e.g. fibroblasts, macrophages, multinucleated giant cells, and plasma cells) will uptake the factors released by MCs in IH [28]. However, the factors being exchanged between these cells remain unclear, as well as the number of activated MCs in IH.

Our results showed that IH in the early and late involuting phases contained more E2 positive MCs compared with proliferative phase specimens (*P* = 0.023 and *P* = 0.006, respectively), suggest that activated MCs are likely release modulators that lead to regression of neovessels in IH. Furthermore, it has been shown that after steroid therapy, the MC number in involuting IH increased by fourfold compared to untreated IH [29].

Interestingly, our results apparently contradict the proangiogenic effect of MCs. However, the fact that not all MCs showed positive staining for E2 in this study, suggest that only certain MCs, activated by E2, are involved in IH regression.

This study had several limitations. First, the number of surgical IH tissue specimens was limited. Second, in this research, all IH specimens resection was followed the IH surgical principles [30]. So, the specimens may not truly represent most IHs, which can be left alone, allowing the natural involution to run its course [30]. Third, the surgical specimens may not truly represent the clinical phase of IH, because sometimes histological features of all three phases may coexist in one surgical IH tissue specimen.

## Conclusions

This study firstly showed the following: (1) E2 and ER-a are expressed on MCs rather than endothelial cells in IH; (2) E2 may regulate MC migration in IH; (3) MCs activated by E2 binding to ER-a are likely involved in IH regression. Although the concentrations of E2 and the molecular mechanisms underlying estrogenic effects on mediator release from activated MCs in IH need further investigation.Our findings suggest a new direction in the study of the mechanisms of estrogen interference of IH development via MCs.

## Abbreviations

DAB: Diaminobenzidine
DAPI: 4′,6-diamidino-2-phenylindole
E2: Estradiol
ER: Estrogen receptor
FGF: Fibroblast growth factor
GLUT-1: Glucose transporter protein-1
H&E: Hematoxylin-eosin
HPF: High power fields
IFN: Interferon
IH: Infantile hemangioma
MCC: Mast cell counts
MCs: Mast cells
PBS: Phosphate buffered saline
TGF: Transforming growth factor
VAs: Vascular anomalies
VEGF: Vascular endothelial growth factor
VMs: Vascular malformations
